# Exploring the Role of Circadian Rhythms in Sleep and Recovery: A Review Article

**DOI:** 10.7759/cureus.61568

**Published:** 2024-06-03

**Authors:** Dev Desai, Aryan Momin, Priya Hirpara, Hemali Jha, Ravi Thaker, Jitendra Patel

**Affiliations:** 1 Internal Medicine, Smt. Nathiba Hargovandas Lakhmichand Municipal Medical College, Ahmedabad, IND; 2 Internal Medicine, Gujarat Medical Education and Research Society Medical College, Vadnagar, IND; 3 Internal Medicine, Integral Institute of Medical Sciences and Research Centre, Lucknow, IND; 4 Physiology, Dr. Narendra Dharmsinh Desai Faculty of Medical Science and Research, Dharmsinh Desai University, Nadiad, IND; 5 Physiology, Gujarat Medical Education and Research Society Medical College, Vadnagar, IND

**Keywords:** health, rem sleep, metabolic effects, circadian rhythm, sleep hygiene

## Abstract

Sleep is essential for every living organism. Humans spend about one-third of their lives sleeping. Sleep has been studied extensively, and the role of sleep in psychological, mental, and physical well-being is established to be the best. The rhythm of the brain between wakefulness and sleep is called the circadian rhythm, which is mainly controlled by melatonin and the pineal gland. The imbalance of this rhythm can lead to devastating effects on health. Vigorous workouts close to bedtime can interfere with falling asleep. Meal timing and composition can significantly affect sleep quality. It is advised to avoid large meals, caffeine, and alcohol before bedtime. Heavy meals close to bedtime can lead to poor sleep and hormone disruption. By following these guidelines enumerated in the article, individuals can improve sleep quality and overall health. Sleep cycles, especially rapid eye movement sleep, have a profound influence on mental and physical health. Adhering to recommended sleep practices enhances bodily restoration, fortifies the immune system, and upholds metabolic equilibrium. Sleep hygiene aligned with circadian rhythms is crucial for disease prevention and well-being. Healthcare professionals should prioritize sleep optimization strategies for patient care and public health.

## Introduction and background

Sleep is an absolute necessity for every living organism. Humans spend about a third of their lives sleeping. Every organism, small or big, requires sleep or a dormancy period. It helps cells remove the toxic metabolites and increases life expectancy and quality. Advanced organisms develop a rhythm with their surrounding nature and the sun-moon cycle called the circadian rhythm.

Circadian rhythms, the inherent 24-hour cycles in our brains that regulate patterns of alertness and sleepiness, respond to the variations in light encountered in our environment, fundamentally influencing a wide range of vital physiological processes. These include our sleep-wake cycles, memory consolidation, metabolic regulation, hormonal balance, and other critical bodily functions. Furthermore, disruptions in these rhythms, due to factors such as exposure to blue light, changes in melatonin and cortisol levels, or conditions such as jet lag and insomnia, can severely impact health, increasing the risk of chronic diseases such as diabetes, obesity, and seasonal affective disorder, as well as various sleep disorders [1]. Such disturbances underscore the intricate relationship between circadian rhythms and our overall well-being, emphasizing the importance of maintaining these natural cycles.

This review delves into the science behind sleep cycles, the restorative power of deep sleep, and the consequences of sleep deprivation, while also exploring the roles sleep plays in physical healing, mental health, and the prevention of diseases [2]. By highlighting the critical role of the suprachiasmatic nucleus and the master clock regulating this rhythm, we aim to provide insights into how optimized sleep hygiene, attention to melatonin production, and lifestyle adjustments can improve sleep quality, thereby enhancing health and preventing sleep-related disorders. Through an understanding of these mechanisms, readers will grasp the profound impact circadian rhythms have on bodily restoration and the potential strategies for mitigating the adverse effects of sleep disturbances [3].

## Review

Science of sleep

Circadian rhythms, intrinsic to our biological makeup, orchestrate a symphony of bodily functions over a 24-hour cycle, profoundly influencing our sleep-wake patterns and overall health. These rhythms, emanating from the brain’s suprachiasmatic nucleus, dictate not only our periods of sleepiness and alertness but also regulate critical functions such as hormone secretion, body temperature, and metabolism.

Circadian rhythms are endogenously generated and are synchronized with the 24-hour day, helping organisms adapt to the solar cycle. In humans, these rhythms are calibrated by the light-dark cycle, with light serving as the primary cue to reset the brain’s circadian clock located in the suprachiasmatic nucleus. This master clock is instrumental in coordinating all circadian clocks throughout the body, ensuring a harmonized physiological rhythm.

The regulation of sleep involves two interacting processes: a homeostatic sleep drive that builds the longer we are awake and dissipates during sleep, and a circadian process that modulates the timing of sleep and wakefulness. These processes ensure that sleep occurs at the optimal time of the biological night, aligning sleep architecture with the body’s internal clock.

**Figure 1:**
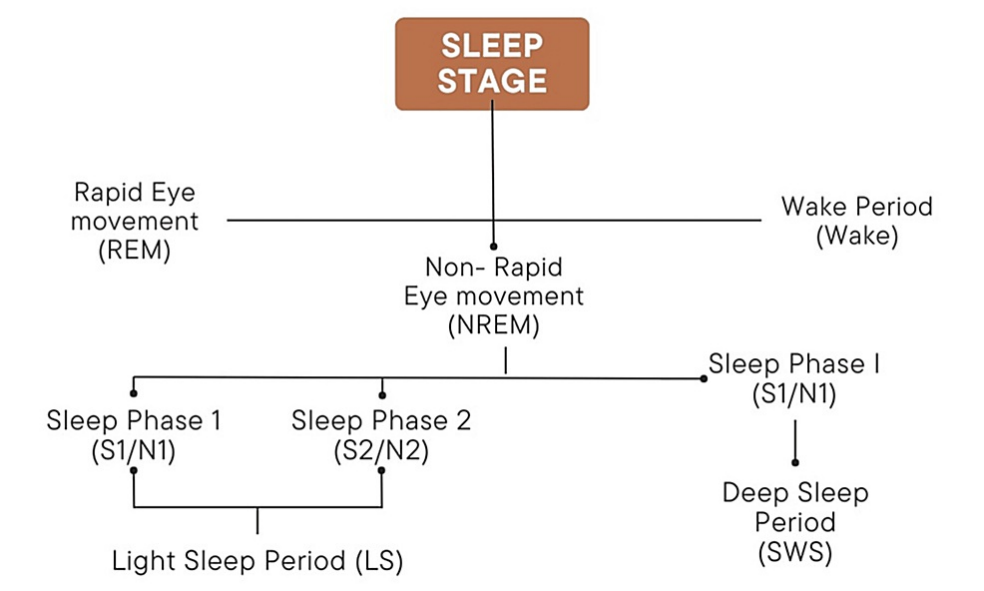
Stages of sleep. Created by the authors.

Sleep is not a uniform state but is composed of several cycles, each consisting of non-rapid eye movement (NREM) sleep and rapid eye movement (REM) sleep, which are fundamentally different in terms of brain activity and physiological functions (Figure 1) [4-6].

NREM Sleep

Stage 1 (S1/N1) is the transition from wakefulness to sleep. Stage 2 (S2/N2) is light sleep before deeper sleep stages. Stage 3 (S3/N3) is deep sleep, crucial for physical restoration and health.

REM Sleep

REM sleep occurs for approximately 90 minutes and is characterized by active brain patterns, eye movement, and dreaming. It is essential for cognitive functions such as memory consolidation and mood regulation.

Disruptions in circadian rhythms, whether from lifestyle factors or environmental cues such as artificial light, can lead to significant health issues, including sleep disorders, metabolic syndrome, and mood disturbances. The precise timing of the circadian clock is crucial for determining the quality and restorative value of sleep, influencing everything from cellular repair to neuroplasticity.

Maintaining a regular sleep schedule and managing light exposure are critical for aligning the circadian rhythm. Exposure to natural light during the day and minimizing blue light exposure from screens before bedtime can help reinforce the natural circadian cycle, enhancing sleep quality and overall well-being.

By understanding and respecting our circadian rhythms and their profound impact on sleep, we can take actionable steps toward improving our health and vitality.

Healing power of deep sleep

Deep sleep, or slow-wave sleep, is critical for physical restoration and recovery. During this phase, the body undergoes significant regenerative processes which are essential for health and well-being. Growth hormone, predominantly secreted during deep sleep [7], plays a pivotal role in tissue growth and muscle repair. This hormone aids in cellular repair and regeneration, helping to counteract the wear and tear experienced during waking hours.

The immune system also benefits markedly from deep sleep. Studies have shown that immune function is enhanced during this sleep phase [8], as the production of cytokines increases. These cytokines are crucial for fighting infections and inflammation, thus supporting the body’s natural defense mechanisms.

Moreover, deep sleep contributes to the detoxification of the brain. The lymphatic system, active primarily during deeper sleep stages [2], facilitates the clearance of metabolic waste products from the central nervous system. This process is vital for maintaining neurological health and function, as it helps prevent the buildup of neurotoxic waste that can contribute to neurodegenerative diseases.

Cardiovascular health is another beneficiary of deep sleep. During this restorative sleep stage, heart rate and blood pressure decrease, which provides an opportunity for the cardiovascular system to recover from the day’s stresses. This reduction in cardiovascular workload during deep sleep is associated with better heart health and a reduced risk of heart disease.

By understanding the profound impact of deep sleep on physical health, individuals can appreciate the necessity of prioritizing sleep quality and duration in their daily lives to support their body’s natural healing and maintenance processes.

How sleep enhances physical healing

Sleep plays a critical role in the physical healing process, acting as a restorative period during which the body can repair and rejuvenate itself. The body performs critical functions such as tissue growth and repair during sleep, which is essential for recovering from daily wear and tear as well as from more significant physical injuries. Sleep facilitates the clearance of metabolic waste products from the brain, owing to the activation of the lymphatic system. This process is crucial for maintaining long-term cognitive health and function. Energy stores are replenished during sleep, providing essential fuel for brain and body functions in the upcoming day. Creating a restful sleeping environment can greatly enhance the quality of sleep, particularly during periods of recovery. Factors such as noise levels, light exposure, and comfort can all influence sleep quality. Insufficient or disrupted sleep can hinder the efficient recovery of physiological functions, such as muscle repair and brain function, and decrease overall well-being [9].

The recovery of metabolic balance and the reduction of stress levels are significantly influenced by sleep. The timing of sleep, activities before sleep, and overall metabolic status contribute to how effectively sleep can promote healing. Incorporating a focus on sleep hygiene and creating a conducive sleeping environment is essential for maximizing the healing benefits of sleep [10]. By understanding and harnessing the power of sleep, individuals can significantly improve their physical health and accelerate recovery processes.

Dreams serve as a profound connection to the subconscious mind, providing insights that are often not accessible during waking hours. They can act as a therapeutic tool, particularly in understanding and resolving deep-seated emotional issues. Dreams allow an individual to engage with the subconscious mind, which can be instrumental in addressing unresolved issues. This engagement often manifests through both recurring and unique dreams offering valuable insights that might remain unrecognized in conscious thought [11]. By presenting new viewpoints or revisiting troubling situations, dreams can offer fresh perspectives that contribute to emotional healing. This process allows individuals to confront problems or situations in a safe, subconscious environment, potentially leading to resolution and personal growth. Interestingly, nightmares can function as a form of exposure therapy. They repeatedly expose the dreamer to their fears, which can help desensitize their emotional response over time, potentially reducing the impact of those fears in waking life [12]. However, the healing potential of dreams can be significantly hampered by disrupted sleep patterns. Conditions such as post-traumatic stress disorder (PTSD), mood disorders, and chronic insomnia can alter dreaming patterns, sometimes eliminating the beneficial effects of dreams or even exacerbating psychological distress. While dreams can raise awareness about emotional and psychological issues, they do not directly heal trauma. Some believe dreams do not affect the healing process [13]. Healing from trauma involves a conscious, deliberate process that includes time, patience, and active self-care measures [14]. To effectively address and heal from trauma, incorporating various healing modalities can be beneficial. Techniques such as mindfulness meditation, therapeutic interventions, and even culturally specific practices such as shamanic ceremonies can complement the insights gained from dreams. The choice of healing methods should align with an individual’s preferences and compatibility, ensuring a holistic approach to mental health and well-being [15,16].

Role of sleep in mental health

Sleep is fundamental to mental health, acting as a crucial period for brain restoration and emotional regulation. The relationship between sleep and mental health is profound and complex, influencing various psychological states and disorders.

Sleep deprivation can lead to a wide range of mental health issues. Individuals experiencing insufficient sleep often face heightened levels of anxiety, depression, and paranoia. The emotional instability can manifest as mood swings, impulsive behavior, and even suicidal thoughts. Moreover, medical students, often subjected to extreme academic pressures and erratic schedules, commonly report poor sleep quality, which further exacerbates stress and anxiety [17].

The brain undergoes crucial processing and recovery during sleep, which is essential for cognitive functions such as memory, focus, and decision-making. Lack of adequate sleep impairs these abilities, leading to decreased learning capacity, poor concentration, and delayed reaction times. Emotional disturbances such as increased irritability, impulsivity, and a general decline in mood regulation are also significant [18-20].

Persistent sleep problems are strongly linked with the development of serious mental health conditions. Research indicates a direct correlation between sleep disturbances and the prevalence of disorders such as depression, bipolar disorder, anxiety disorders, PTSD, and eating disorders [21]. Addressing insomnia and other sleep issues early can be crucial in preventing or mitigating these conditions.

REM sleep, the phase associated with vivid dreaming, plays a critical role in emotional and cognitive processing. Alterations in REM sleep patterns have been observed in various mood disorders, including depression. Studies suggest that selective REM sleep deprivation may relieve depressive symptoms in some cases, although the exact mechanisms remain under investigation. This phase of sleep is also pivotal for the consolidation of emotional memories, which can affect mood and mental health [18-20].

Improving sleep quality has shown significant positive effects on various aspects of mental health. Enhancements in mood, anxiety levels, and stress are notable, with specific improvements in depression scores. Effective management of sleep disorders, such as sleep apnea, has also been shown to alleviate depressive symptoms and restore optimal mental functioning.

The intricate interplay between sleep and mental health underscores the necessity of maintaining healthy sleep patterns. By fostering better sleep hygiene and addressing sleep disturbances proactively, individuals can significantly enhance their mental well-being and overall quality of life.

REM sleep, a critical phase of our sleep cycle, plays a vital role in mental health by facilitating complex processes that enhance emotional stability and cognitive functions [22]. This stage is marked by unique neurochemical and physiological activities that significantly affect our mental well-being.

REM sleep is distinct in its neurochemical profile, characterized by the absence of the anxiety-inducing molecule noradrenaline. This creates a neurochemically safe environment that allows for the reprocessing of emotional memories without the interference of stress-related chemicals [23]. Additionally, during REM sleep, there is a specific pattern of neurochemical changes, including increased activity in subcortical cholinergic systems and decreased activity in aminergic systems, which modulate emotions effectively.

The processing and consolidation of emotional memories are significantly enhanced during REM sleep. The amygdala, a key brain structure involved in emotional processing, plays a crucial role during this sleep stage. It facilitates the initial acquisition and subsequent processing of emotional memories by influencing other critical areas such as the hippocampal complex [24]. This process is crucial for emotional regulation and the maintenance of mental health.

REM sleep has been shown to foster creativity and enhance problem-solving abilities. This sleep stage encourages the brain to fuse and blend individual memories in abstract and highly novel ways, which can lead to insights and solutions that elude us during waking hours. By extracting overarching rules and commonalities from disparate pieces of information, REM sleep supports creative thinking and complex problem-solving.

Dreaming, a phenomenon primarily occurring during REM sleep, plays a therapeutic role in managing emotional reactivity. The emotional content of dreams, paired with reduced brain noradrenaline levels, helps de-escalate emotional responses and may aid in healing emotional wounds. This process is supported by low stress hormone levels in the body during REM sleep, further facilitating a conducive environment for emotional processing.

Advanced neuroimaging techniques have revealed that REM sleep is associated with elevated activity in several brain regions critical for emotion regulation, including the pontine tegmentum, thalamic nuclei, occipital cortex, mediobasal prefrontal lobes, and affect-related areas such as the amygdala and hippocampus [23]. These insights underscore REM sleep’s complex and dynamic functional anatomy, highlighting its importance in emotional and psychological health.

Understanding the multifaceted role of REM sleep in mental health not only highlights its importance but also emphasizes the need for sleep quality management to support emotional and psychological well-being.

Sleep and brain function

Sleep is fundamental to brain function, impacting everything from cognitive abilities to emotional stability. The intricate relationship between sleep and brain function is underscored by various physiological and neurological processes that occur during different sleep stages.

The brain relies on sleep to perform critical functions such as memory consolidation, cognitive processing, and detoxification. During sleep, neurons communicate intensively, and the brain eliminates toxins that accumulate during waking hours, which is essential for maintaining long-term cognitive health [25]. Sleep regulates neurotransmitters that affect mood and cognitive functions. Proper sleep helps maintain the balance of chemicals such as serotonin and dopamine, which play a key role in mood regulation and mental clarity. Sleep is crucial for the consolidation of memories. The processes that transfer and solidify new learning occur predominantly during sleep, particularly during REM and deep sleep stages [26]. Sleep promotes brain plasticity, which is the brain’s ability to adapt to new information and experiences. This aspect of brain function is vital for learning new skills and for overall cognitive flexibility.

Insufficient sleep can severely impair brain function, affecting everything from complex decision-making to simple daily tasks. Lack of sleep affects the brain’s ability to process information and can significantly impair judgment and memory, making decision-making more difficult. Sleep deprivation can disrupt the central nervous system, affecting how the brain sends and processes information, leading to decreased alertness and cognitive performance [18-20].

Circadian rhythms play a significant role in optimizing brain function by regulating sleep patterns that are aligned with our environmental cues. Through the suprachiasmatic nucleus, the hypothalamus controls behavioral rhythms based on light exposure, influencing when we sleep and wake, and impacting our cognitive functions [27]. The brainstem, which includes the pons, medulla, and midbrain, plays a critical role in controlling transitions between wake and sleep, ensuring that these transitions support optimal brain function.

Promoting good sleep hygiene can significantly improve brain function and overall mental health. Establishing a consistent sleep routine and optimizing the sleep environment can enhance the quality of sleep, thereby supporting better brain function and reducing the risk of sleep-related cognitive impairments [28-30]. Regular physical activity has been shown to improve sleep quality and increase adenosine levels in the brain, which helps regulate fatigue and supports cognitive functions.

This review aims to identify biomarkers that predict human performance under conditions where circadian and sleep homeostatic systems are challenged, highlighting the critical nature of sleep in supporting brain function and overall health. Understanding these dynamics allows for better management of sleep patterns, ultimately enhancing cognitive, emotional, and physiological health [31,32].

Impact of sleep deprivation

Sleep is a fundamental component of overall health, influencing various physiological systems, including the immune system and metabolism. Understanding the interplay between sleep, immune function, and metabolic processes is crucial for maintaining health and preventing disease.

The functionality of the central nervous system, crucial for processing and transmitting information, is significantly impaired by sleep deprivation. Insufficient sleep can impair memory and make decision-making more difficult due to affected judgment and cognitive processing. Chronic sleep deprivation can lead to difficulties in learning new things and coordinating actions, increasing the risk of accidents [18-20].

Deprivation of REM sleep can have detrimental effects on mental health, impairing the consolidation of emotional memories and exacerbating negative emotional reactions. It has been shown that lack of REM sleep can lead to increased irritability, affective volatility, and a skewed memory recall that favors negative over positive or neutral memories [23]. Furthermore, sleep deprivation disrupts the functional connectivity between the amygdala and the medial prefrontal cortex, leading to heightened reactivity to negative emotional stimuli.

Sleep deprivation poses serious risks to cardiovascular health and the body’s ability to fight infections. Sleep loss can prevent the normal drop in blood pressure during sleep and trigger inflammation, increasing the risk of heart disease and stroke. Chronic conditions such as hypercholesterolemia, hypertension, and diabetes are also linked to insufficient sleep [33,34].

The immune system benefits significantly from sleep, which enhances its ability to defend against infections and inflammation. During sleep, the body undergoes several processes that bolster immune defense. Sleep promotes the production of cytokines, proteins that are critical in controlling the immune response [35]. These cytokines help the body combat infections and inflammation during sleep. Sleep facilitates the movement of T cells to lymph nodes, which is essential for effective immune response. This redistribution helps the body respond more effectively to pathogens. Just like cognitive memory, the immune system’s memory is strengthened during sleep, enhancing its ability to recognize and respond to antigens more effectively [36].

Sleep plays a pivotal role in metabolic health, influencing various functions that are critical for energy use and overall metabolic balance. During sleep, important metabolic hormones are released [37]. These include hormones that help regulate glucose levels and appetite, significantly impacting body weight management and metabolic health. Sleep affects the regulation of leptin and ghrelin, hormones that control feelings of hunger and fullness. Adequate sleep helps maintain a balance between these hormones, thereby supporting healthy eating behaviors and weight management. Sleep contributes to the body’s basal metabolic rate, which is the rate at which the body uses energy while at rest. Good sleep helps maintain a healthy metabolic rate, which is crucial for energy balance and weight control [38-40].

Circadian rhythms, the body’s internal biological clock, are deeply intertwined with both immune and metabolic functions. They help regulate the timing of sleep and influence the effectiveness of the immune response and metabolic processes [41,42]. The circadian system regulates the timing of immune functions, with certain immune responses being more effective at specific times of the day. These rhythms also dictate metabolic functions, such as glucose regulation and energy utilization, aligning these processes with day-night cycles to optimize health [43-45].

Lack of adequate sleep can severely disrupt both immune and metabolic functions, leading to a range of health issues. Sleep deprivation reduces the effectiveness of the immune system, making the body more susceptible to infections and diminishing the response to vaccinations [46]. Insufficient sleep can lead to metabolic imbalances, increasing the risk of obesity, diabetes, and other metabolic syndromes. By prioritizing sleep and aligning it with natural circadian rhythms, individuals can enhance their immune and metabolic health, reducing the risk of disease and improving overall well-being [3].

The broader implications of sleep deprivation extend to an increased risk of both acute and chronic illnesses. Individuals who consistently get less than seven hours of sleep are significantly more likely to contract common illnesses such as colds [47]. A pattern of poor sleep is linked to serious health issues, including heart attacks, cognitive decline, and early death. Sleep deprivation can also lead to neurodegenerative conditions by decreasing the levels of protective proteins in the brain, leading to neuronal death. Understanding and addressing sleep deprivation is crucial for maintaining long-term health and preventing a range of chronic conditions. By prioritizing sleep and managing lifestyle factors that influence sleep quality, individuals can significantly improve their overall well-being and reduce the risk of numerous health complications [48].

Improving sleep quality for better health

Good sleep patterns facilitate wiser food choices, while a balanced diet enhances sleep quality. Ensuring an intake of nutrients that support the sleep cycle, such as magnesium and tryptophan, can lead to improved sleep quality. Aligning sleep schedules with natural circadian rhythms enhances overall health by improving sleep quality and increasing daytime alertness. The American Heart Association advises adults to aim for seven to nine hours of sleep nightly to support optimal heart and brain health. This recommendation aligns with general guidelines that suggest adults need at least seven hours of sleep per night. Sleep duration requirements vary significantly with age. Adults typically need seven to eight hours, teenagers require about nine hours, young children need at least 10 hours, and infants up to 16 hours of sleep per day.

To enhance sleep quality, it is crucial to establish a consistent bedtime routine and create a sleep-conducive environment. It is important to stick to regular sleeping and waking time, even on weekends, to regulate the body’s clock [30]. It is crucial to ensure the bedroom is quiet, dark, and cool and to remove electronic devices that emit blue light and can disrupt sleep. Avoiding large meals, caffeine, and alcohol before bedtime helps prevent disruptions in sleep quality. Engaging in regular exercise, but not close to bedtime, promotes better sleep [49].

Supplements such as melatonin help regulate sleep patterns and improve sleep onset. Other natural supplements such as valerian root and lavender may also aid in relaxation and better sleep quality. Reducing irregular or long daytime naps and avoiding caffeine late in the day can further support night-time sleep quality [50].

Sleep plays a pivotal role in disease prevention, influencing various health outcomes and reducing the risk of chronic illnesses. Understanding how adequate sleep acts as a preventive measure can empower individuals to make informed decisions about their health routines [46,51].

Engaging in regular exercise during youth can significantly reduce the risk of developing chronic diseases in later life, such as cardiovascular disorders and metabolic syndrome. A lack of sufficient sleep is associated with an increased risk of developing severe health issues, including heart disease, high blood pressure, obesity, and stroke. Inadequate sleep can disrupt normal nocturnal blood pressure patterns, leading to persistent hypertension and inflammation [52]. This condition heightens the risk of developing cardiovascular diseases, including heart attacks and strokes. The quality and duration of sleep are critical predictors of hemoglobin A1c levels, a key indicator of glycemic control in individuals with type 2 diabetes. Proper sleep helps maintain optimal blood sugar levels, thereby preventing the onset of diabetes-related complications. Individuals suffering from sleep apnea are at an elevated risk of developing various cardiovascular conditions. These include hypertension, stroke, coronary artery disease, and cardiac arrhythmias, underscoring the need for effective management of sleep disorders to prevent these serious health outcomes [18-20].

By implementing these strategies and paying attention to both the physical environment and personal habits, individuals can significantly improve their sleep quality, which is essential for overall health and well-being [53]. By prioritizing sleep and integrating it with healthy lifestyle choices such as regular physical activity, individuals can significantly enhance their preventive care strategies, reducing the risk of chronic diseases and improving overall health.

Nutrition and exercise for better sleep

Adequate nutrition plays a critical role in maintaining the health and academic performance of individuals, particularly those in high-stress environments such as medical students [17]. A balanced diet rich in vegetables and fruits provides essential vitamins and minerals that contribute significantly to improved sleep quality. Notably, diets such as the Mediterranean and DASH diets are beneficial, promoting better sleep through their rich nutrient profiles. It is generally advisable to prioritize a heart-healthy diet encompassing a variety of fiber, produce, and protein, while minimizing the intake of saturated fats, rather than focusing on specific nutrients [54]. Engaging in regular physical activity is profoundly beneficial for night-time rest. Exercise, particularly when performed during daylight hours, is one of the best ways to ensure a good night’s sleep. It is recommended to include at least 20 minutes of physical activity daily as part of a sleep-enhancing routine. However, it is crucial to avoid vigorous workouts close to bedtime as they might interfere with the ability to fall asleep. The timing and composition of meals can significantly affect sleep quality. It is advised to avoid large meals, caffeine, and alcohol before bedtime, as these can disrupt sleep by causing discomfort, increasing metabolism, and altering hormone levels. Specifically, consuming heavy meals close to bedtime can lead to poor sleep and hormone disruption, further emphasizing the importance of meal timing in sleep hygiene.

By incorporating these nutritional and physical activity guidelines into daily routines, individuals can significantly improve their sleep quality, which, in turn, enhances overall health and well-being [55].

## Conclusions

Through an extensive analysis of the interconnected roles of sleep cycles, REM sleep, and their profound influence on mental and physical health, this article has underscored the intrinsic value of circadian rhythms and sleep hygiene. By delineating the mechanisms through which sleep enhances bodily restoration, fortifies the immune system, and upholds metabolic equilibrium, we have elucidated the significance of adhering to recommended sleep practices. These practices not only mitigate the risk of chronic diseases but also optimize cognitive and emotional function, thereby elevating overall quality of life. In the medical fraternity, where a nuanced understanding of sleep’s multifaceted benefits is essential, the synthesis of research presented herein serves as a clarion call to prioritize sleep. The alignment of sleep hygiene with circadian rhythms emerges as a pivotal strategy for disease prevention and the promotion of holistic well-being. It is incumbent upon healthcare professionals to advocate for and integrate sleep optimization strategies within patient care protocols to harness the therapeutic potential of sleep. This comprehensive approach ensures the enhancement of patient outcomes and the broader public health landscape.
